# Feasibility study of dual parametric 2D histogram analysis of breast lesions with dynamic contrast-enhanced and diffusion-weighted MRI

**DOI:** 10.1186/s12967-018-1698-x

**Published:** 2018-11-23

**Authors:** Yanqiong Chen, Bin Wu, Hui Liu, Dan Wang, Yajia Gu

**Affiliations:** 1 Fudan University Shanghai Cancer Center, No. 270, Dong’an Rd, Shanghai, 200032 China; 2 Imaging Technology (Shanghai), Shanghai, China

**Keywords:** DCE, DWI, MRI, Multiparametric histogram analysis, Breast

## Abstract

**Background:**

This study aimed to investigate the diagnostic value of a dual-parametric 2D histogram classification method for breast lesions.

**Methods:**

This study included 116 patients with 72 malignant and 44 benign breast lesions who underwent CAIPIRINHA-Dixon-TWIST-VIBE dynamic contrast-enhanced (CDT-VIBE DCE) and readout-segmented diffusion-weighted magnetic resonance examination. The volume of interest (VOI), which encompassed the entire lesion, was segmented from the last phase of DCE images. For each VOI, a 1D histogram analysis (mean, median, 10th percentile, 90th percentile, kurtosis and skewness) was performed on apparent diffusion coefficient (ADC) and volume transfer constant (Ktrans) maps; a 2D histogram image (Ktrans-ADC) was generated from the pixelwise aligned maps, and its kurtosis and skewness were calculated. Each parameter was correlated with pathological results using the Mann–Whitney test and receiver operating characteristic curve analysis.

**Results:**

For the Ktrans histogram, the area under the curve (AUC) of the mean, median, 90th percentile and kurtosis had statistically diagnostic values (mean: 0.760; median: 0.661; 90th percentile: 0.781; and kurtosis: 0.620). For the ADC histogram, the AUC of the mean, median, 10th percentile, skewness and kurtosis had statistically diagnostic values (mean: 0.661; median: 0.677; 10th percentile: 0.656; skewness: 0.664; and kurtosis: 0.620). For the 2D Ktrans-ADC histogram, the skewness and kurtosis had statistically higher diagnostic values (skewness: 0.831, kurtosis: 0.828) than those of the 1D histogram (all *P* < 0.05).

**Conclusions:**

The dual-parametric 2D histogram analysis revealed better diagnostic accuracy for breast lesions than single parametric histogram analysis of either Ktrans or ADC maps.

## Background

Dynamic contrast-enhanced (DCE) MRI detects sequential changes in contrast enhancement that can be interpreted for quantitative analysis [1, 2]. Pharmacokinetic parameters based on the Tofts model [3] derived from DCE MRI have shown to have a good correlation with tumor angiogenesis [4, 5], and among these parameters, the volume transfer constant (Ktrans) has potential for differentiating of breast lesions. Malignant tumors tend to have higher Ktrans values than benign tumors [4–7]. However, the reported Ktrans values for breast lesions and the diagnostic accuracy of these values fluctuates with different MRI acquisition environment and postprocessing techniques. One particular issue with DCE MRI of previous studies is a lack of simultaneously high temporal and high spatial resolution DCE sequences due to technological limitations [4, 7–9]. According to the model, higher temporal resolution helps to modify the dynamic curve to be closer to the real physiological situation. Though not directly comparable, studies have verified that high temporal resolution sequences have better diagnostic performances than low temporal resolution sequences [5, 10]. Recently, high temporal resolution can be achieved while spatial resolution is preserved with controlled aliasing in parallel imaging results in higher acceleration (CAIPIRINHA)-Dixon-time-resolved angiography with stochastic trajectories (TWIST)-volumetric interpolated breath-hold examination (VIBE) DCE and this sequence has demonstrated promising results [11, 12].

Diffusion-weighted imaging (DWI) is another widely studied MRI technique based on the assumption that the movement of water molecules is more restricted in malignant tumors [13–16]. The apparent diffusion coefficient (ADC), which is derived from DWI, has shown to be useful for differentiating breast lesions. The reported mean ADC values and their diagnostic value varies depending on different DWI scanning environments [13–18] and image interpretations. Nevertheless, there is substantial overlap of ADC values between benign and malignant lesions. With the introduction of readout-segmented echo-planar imaging (RS-EPI) for breast MR imaging, DWI image quality has significantly improved and lesion conspicuity of RS-EPI DWI is better than that of single-shot echo-planar imaging (SS-EPI) with 3 T MRI [19].

One possible approach is to combine DCE and DWI to obtain better performance in terms of differential diagnosis. Histogram analysis is a mathematical method [12, 20–22] that excels in describing tumor heterogeneity by providing quantitative metrics. With the two state of the art acquisition sequences, CAIPIRINHA-Dixon-TWIST (CDT)-VIBE DCE and readout-segmented diffusion-weighted (RS-DWI) MRI, we proposed a dual parametric 2D histogram to combine Ktrans and ADC in this study and hypothesized that this histogram would perform better than single parametric histogram for differentiating breast lesions.

## Methods

### Patients

The institutional review board approved this prospective study and waived the informed consent requirement. From April 2014 to August 2017, 130 female patients with breast masses palpated or indicated by mammography or ultrasound were scanned with CDT-VIBE DCE and RS-DWI MRI. Fourteen patients were excluded for the following reasons: (a) no obvious lesion was detected on MRI (n = 8), (b) pathology revealed a borderline phyllode tumor (n = 3) or lymphoma (n = 1), and (c) there was incomplete fat suppression on DCE images (n = 2). The enrolled patients underwent resection surgery after MRI examinations. For patients with multiple lesions, the largest one was evaluated. Therefore, the final study cohort included 116 lesions from 116 patients. Morphological features and enhancing patterns were described according to the Breast Imaging Reporting and Data System (BI-RADS).

### MR imaging protocol

All patients underwent breast MRI in the prone position with a 3 T MR scanner (Skyra; Siemens Healthcare, Erlangen, Germany) using a dedicated 16-channel bilateral breast coil. Dynamic imaging was performed first with the prototype CDT-VIBE DCE sequence, which consisted of two sequences: 39 s for T1 mapping (using two flip angles: 2 and 14) and 4.44 min for 40 phases of DCE imaging. The two sequences shared the same spatial resolution and field of view (FOV). Gadolinium–diethylene triamine pentaacetic acid (Gd-DTPA) (Magnevist, Bayer Healthcare, Berlin, Germany) was administered intravenously with a power injector at the beginning of the 4th phase at a dose of 0.1 mmol/kg of body weight and a rate of 2 mL/sr. Axial (5 min after contrast injection) and sagittal (7 min after contrast injection) contrast-enhanced images were also obtained for routine clinical diagnosis. Then, the RS-DWI sequence was performed (17 min after contrast injection). Detailed parameters of the sequences are listed in Table 1.

**Table 1:** MRI sequence parameters

	DCE	DWI
Sequence	TWIST-VIBE	RS-EPI
Orientation	Axial	Axial
TR/TE (ms)	5.4/2.46	6300/54
Flip angle	9	180
FOV (mm^2^)	360 * 360	340 * 242
Slice thickness (mm)	2.5	5.5
No. of sections	60	28
Matrix	384 * 384	202 * 144
Temporal resolution (s)	5.6	NA
b value (s/mm^2^)	NA	50, 800
Scanning time (min)	4.44	4.45

*NA* not applicable

### Image interpretation

Pharmacokinetic analysis was performed using the DCE software package (Tissue 4D, version: syngo MR D13, Siemens Healthcare, Germany). After motion correction of the 40 phases of DCE images, pharmacokinetic parameters were calculated based on the two-compartment Tofts model [3] with a population average arterial input function (slow type) provided by Tissue 4D.

ADC maps were generated after DWI scans and exported to a prototype registration package based on the Insight Toolkit (ITK). Each ADC map was rigidly registered and resliced to be aligned with the last phase of the DCE images. The volume of interest (VOI) was segmented using the active contour segmentation mode of the ITK-SNAP software (version 3.2, University of Pennsylvania, Philadelphia, USA) [23] to cover the entire breast lesion on each slice. Cystic areas, vessels, calcifications and artifacts were removed. All the VOIs were drawn by one junior radiologist and confirmed by another senior radiologist.

For each ADC and Ktrans 1D histogram, the following parameters were calculated: mean, median, 10th percentile, 90th percentile, kurtosis and skewness. A normalized 2D histogram (Ktrans-ADC) map was calculated from the registered and resliced Ktrans and ADC maps for each VOI in a prototype dual-parameter mapping package, and its kurtosis and skewness were calculated. Each parameter was correlated with pathological results.

### Statistical analysis

Histogram parameters and other continuous data are expressed as the mean (± standard deviation) and were compared with the Mann–Whitney test. Categorical data was compared with the Chi-squared test. Receiver operating characteristic (ROC) curve analysis was used to evaluate the effectiveness of histogram parameters for differentiating breast lesions. The optimal thresholds were chosen at the point closest to the top-left part of the plot with perfect sensitivity or specificity. Two ROC curves were compared using the DeLong method. Statistical analyses were performed with MedCalc (Version 16.8, MedCalc Software, Mariakerke, Belgium). *P* values of less than 0.05 were deemed to indicate statistical significance.

## Results

### Lesions

Pathological results revealed that 72 lesions were malignant tumors, comprising 62 invasive ductal carcinomas (IDCs), 7 ductal carcinomas in situ (DCIS), 1 invasive lobular carcinoma (ILC), 1 solid papillary carcinoma (SPC), and 1 myoepithelial carcinoma (MEC), while 44 lesions were benign breast diseases, comprising 21 fibroadenomas, 10 intraductal papillomas (IPs), 6 cases of benign adenosis, 5 benign phyllodes tumors and 2 cases of mastitis. The lesions varied in long diameter from 9 to 74 mm and there was no significant difference in lesion size between the two groups. The detailed morphological features and enhancing patterns are listed in Table 2.

**Table 2:** Morphological features of benign and malignant breast lesions

	Benign	Malignant	*P* value
No. of lesions	44	72	
Age (years)	39.54 ± 11.75	50.46 ± 11.54	< 0.001
Distribution			0.974
Mass	40	64	
Non-mass enhancement	4	8	
Diameter (mm)	23.08 ± 13.49	23.89 ± 13.57	0.451
Shape (only mass)			0.214
Oval/round	27	34	
Irregular	13	30	
Margins (only mass)			< 0.001
Spiculated	17	57	
Circumscribed	23	7	
Enhancement pattern			< 0.001
Homogeneous	13	12	
Heterogeneous	19	47	
Rim	5	13	
Dark internal septations	7	0	

Data are reported as the number of lesions and values of parameters (mean ± standard deviation)

### Histogram analysis

**Fig. 1:**
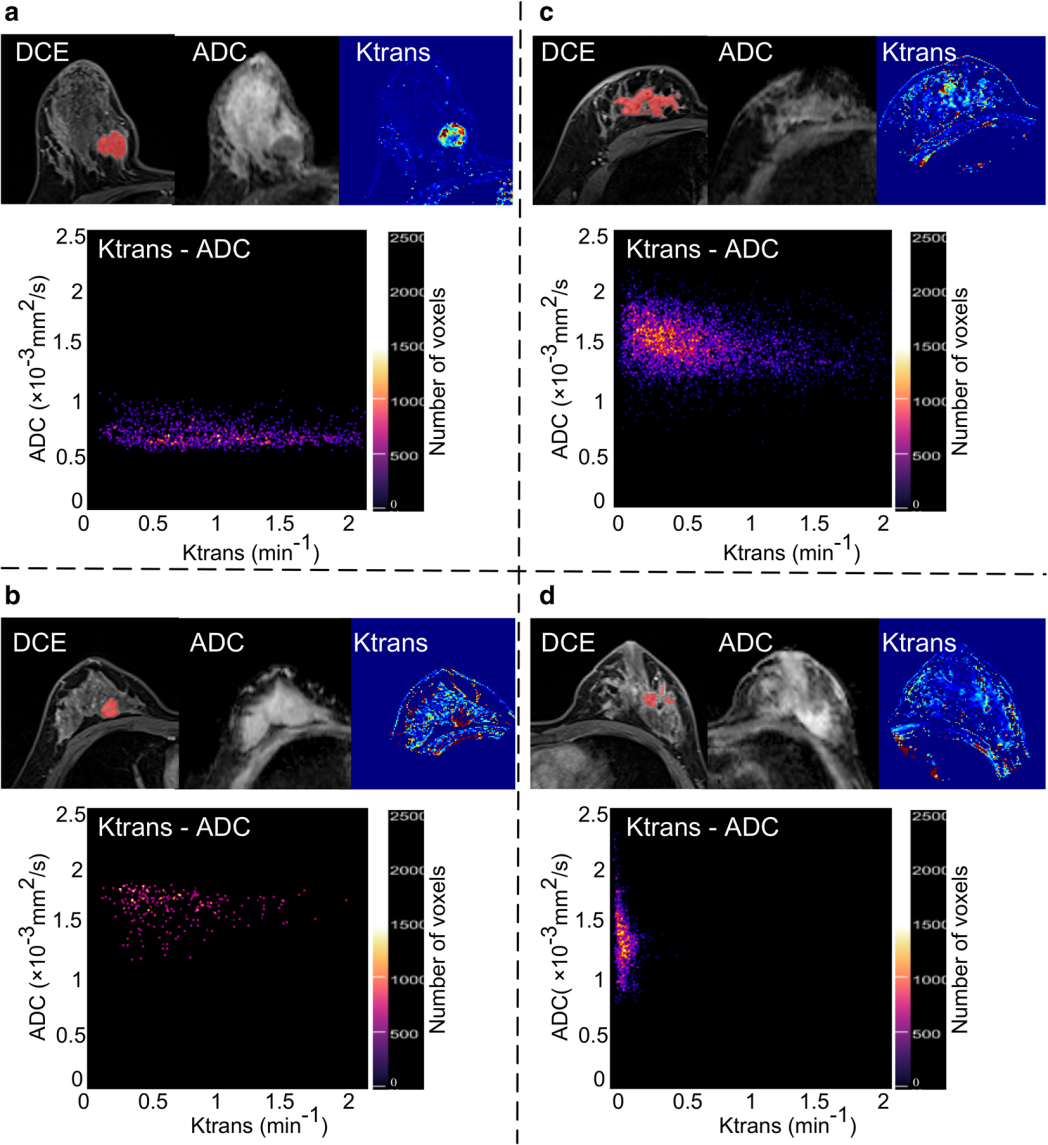
Scatter plot of the 2D histogram. The last phase of DCE imaging, realigned ADC, Ktrans, and Ktrans-ADC 2D histogram map of four malignant and benign breast lesions (**a**: invasive ductal carcinoma, **b**: fibroadenoma, **c**: ductal carcinoma in situ, and **d**: mastitis) are shown in this figure, in which **a**, **b** show the case that Ktrans alone fails to differentiate between malignant and benign lesions, but ADC resolves the ambiguity, while **c**, **d** show the opposite case that ADC fails to differentiate between malignant and benign lesions, but Ktrans resolves the ambiguity

**Fig. 2:**
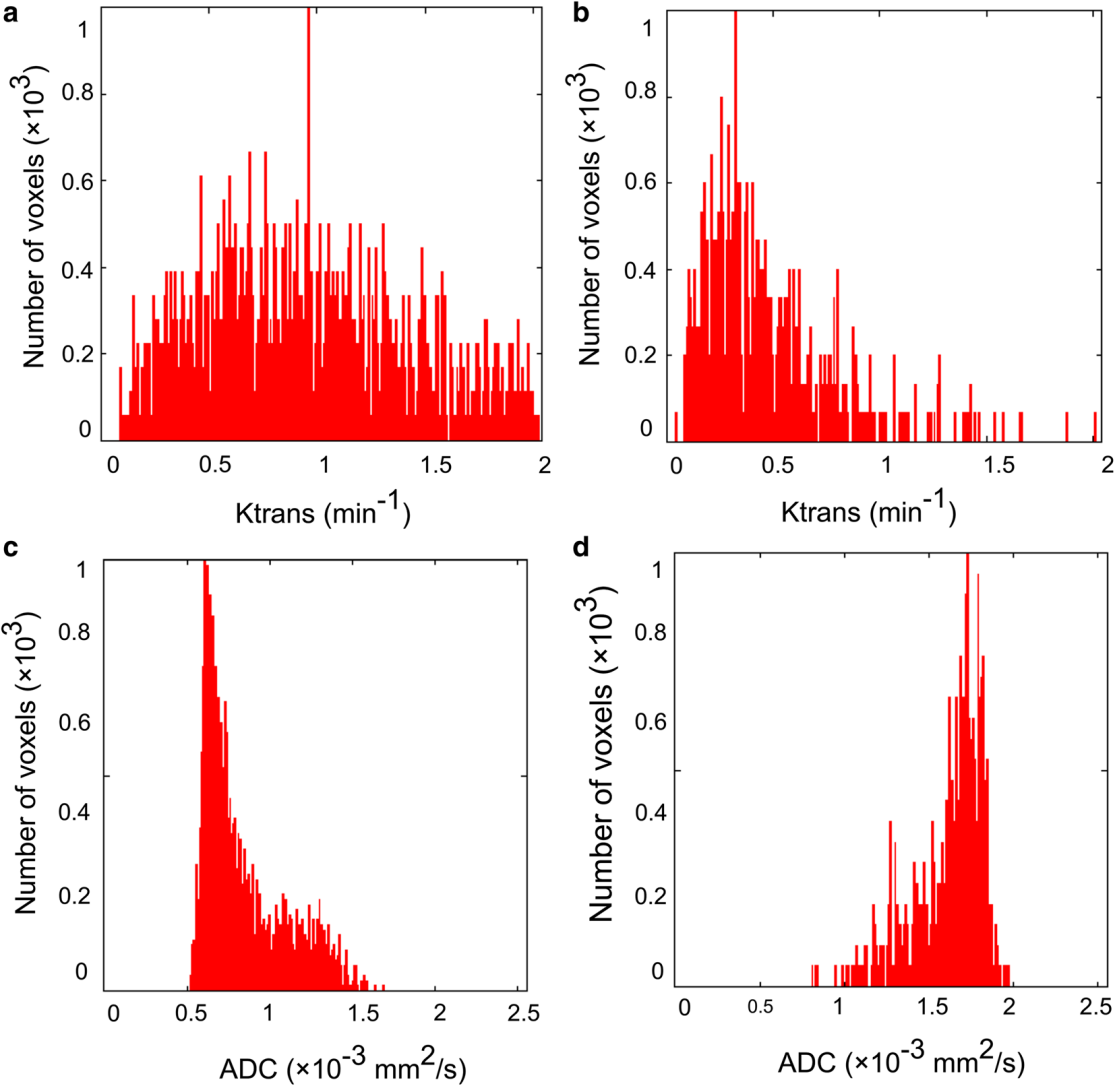
Single parametric histogram. According to the Ktrans and ADC histograms of an invasive ductal carcinoma (**a**, **c**) and a fibroadenoma (**b**, **d**), the kurtosis is (**a** 3.60; **b** 7.56, **c** 2.58; and **d** 5.87), while the skewness is (**a** 1.03; **b** 1.82; **c** 0.84; and **d** − 1.45). For the Ktrans histogram curves, **b** shows a sharper peak and leans more to the left, while for the ADC histogram curves, **d** seems slightly steeper and leans to the right, and **c** leans to the left

Histogram analysis and comparisons between benign and malignant lesions are shown in Table 3. Malignant tumors had higher Ktrans values and lower ADC values than benign tumors. For Ktrans, the mean (*P* < 0.0001), median (*P* = 0.003), 90th percentile (*P* < 0.0001) were significantly different between benign and malignant tumors, and for ADC, the mean (*P* = 0.013), 10th percentile (*P* = 0.024) and median (*P* = 0.006) were significantly different between benign and malignant tumors. When comparing distribution parameters, the kurtosis of Ktrans (*P* = 0.028) and the skewness (*P* = 0.011) of ADC were significantly different between benign and malignant tumors. In the 2D histogram analysis, both skewness (*P* < 0.0001) and kurtosis (*P* < 0.0001) were significantly different between benign and malignant tumors. Figure 1 shows the two situations in which Ktrans and ADC resolved each other’s ambiguity. Figure 2 shows the intuitive differences in skewness and kurtosis in Ktrans and ADC histograms between malignant tumor and benign lesions.

**Table 3:** Histogram analysis of benign and malignant breast lesions

	Benign	Malignant	*P* value
No. of lesions	44	72	
Ktrans (min^−1^)			
Mean	0.34 ± 0.32	0.61 ± 0.29	< 0.001
10th percentile	0.04 ± 0.10	0.04 ± 0.07	0.694
Median	0.22 ± 0.28	0.38 ± 0.30	0.003
90th percentile	0.79 ± 0.66	1.54 ± 0.78	< 0.001
Skewness	2.66 ± 3.48	1.61 ± 0.76	0.124
Kurtosis	30.46 ± 31.53	7.04 ± 6.24	0.028
ADC (10^−3^ mm^2^/s)			
Mean	1.26 ± 0.29	1.12 ± 0.24	0.013
10th percentile	0.67 ± 0.29	0.51 ± 0.22	0.024
Median	1.26 ± 0.30	1.10 ± 0.25	0.006
90th percentile	1.79 ± 0.42	1.87 ± 0.43	0.314
Skewness	− 0.01 ± 0.54	0.33 ± 0.45	0.011
Kurtosis	3.64 ± 1.14	3.17 ± 0.82	0.064
Ktrans-ADC			
Skewness	10.14 ± 6.12	5.54 ± 1.84	< 0.001
Kurtosis	165.59 ± 225.37	43.96 ± 28.85	< 0.001

Data are reported as the values of parameters (mean ± standard deviation)

### ROC curve analysis results

**Fig. 3:**
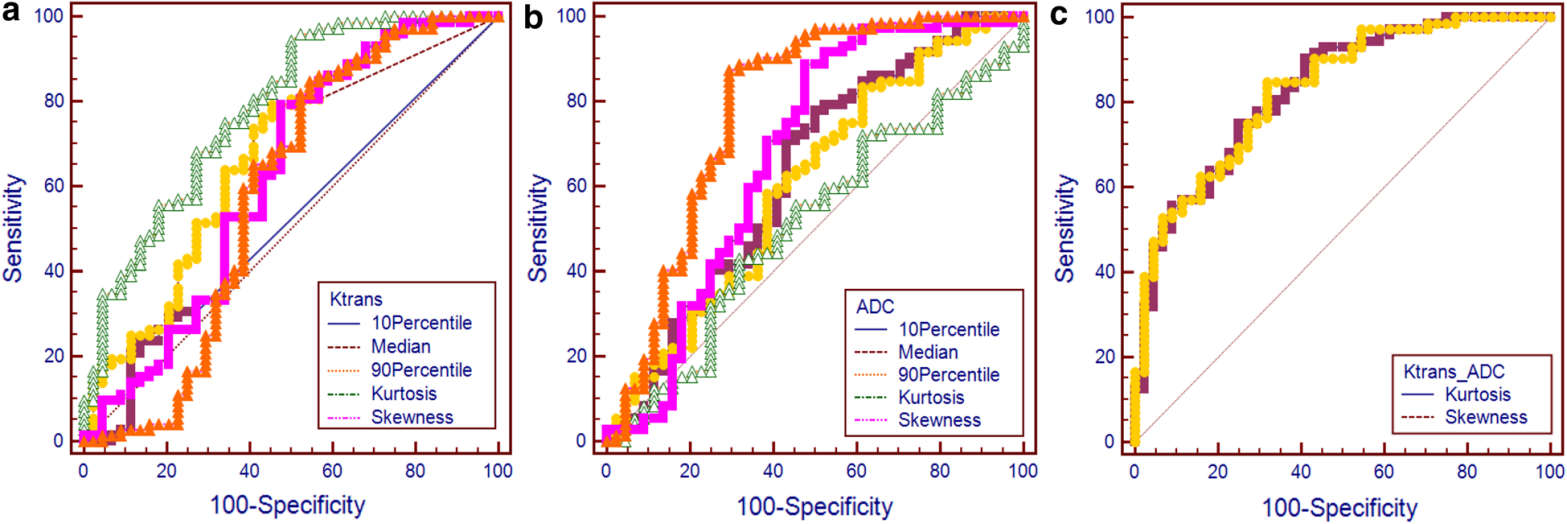
ROC plot. ROC plot of 1D histogram analysis results for Ktrans (**a**) and ADC (**b**), and the kurtosis and skewness derived from the 2D histogram Ktrans-ADC map (**c**) for the entire tumor volume

The ROC curve analysis results are shown in Table 4 and Fig. 3. For Ktrans histograms, the 10th percentile revealed the highest AUC (0.781), while the median of the ADC histogram revealed the highest AUC (0.677). The AUC of the 2D Ktrans-ADC histogram-derived skewness and 2D kurtosis were were 0.831 and 0.828, respectively, and were significantly higher than all of the Ktrans and ADC histogram parameters (all *P* < 0.05), reaching a sensitivity of 68.18% and 75% for skewness and kurtosis, respectively, and a specificity of 84.72% and 75.0% for skewness and kurtosis, respectively.

**Table 4:** ROC curve analysis

	Area under the curve	Threshold	Sensitivity (%)	Specificity (%)
Ktrans-ADC				
Skewness	0.831 (0.796, 0.905)	7.00	68.18	84.72
Kurtosis	0.828 (0.792, 0.904)	55.69	75.0	75.0
Ktrans				
Mean	0.760 (0.660, 0.801)	0.379	72.73	73.61
10th percentile	0.518 (0.429, 0.605)	0.046	77.27	30.56
Median	0.661 (0.557, 0.764)	0.180	59.09	73.61
90th percentile	0.781 (0.693, 0.801)	0.991	72.73	68.06
Skewness	0.585 (0.462, 0.708)	1.647	59.09	65.28
Kurtosis	0.620 (0.508, 0.735)	7.501	52.27	79.17
ADC				
Mean	0.661 (0.566, 0.747)	1.24	76.14	61.54
10th percentile	0.656 (0.561, 0.742)	0.64	80.68	50.00
Median	0.677 (0.583, 0.762)	1.29	84.09	53.85
90th percentile	0.565 (0.469, 0.658)	1.97	46.59	76.92
Skewness	0.664 (0.569, 0.750)	0.04	85.23	46.15
Kurtosis	0.620 (0.524, 0.709)	3.37	71.59	57.69

Data in parentheses are 95% confidence intervals

## Discussion

In this study, we combined the Ktrans derived from DCE and the ADC from DWI to form a 2D histogram and tested its feasibility for differentiating breast lesions. The results showed that the skewness and kurtosis of the 2D Ktrans-ADC histogram were significantly different between malignant and benign breast lesions and had better diagnostic performance than 1D histogram parameters.

Ktrans refers to the coefficient of transendothelial transport of contrast media from the vascular compartment to the tissue interstitium and is correlated to blood flow and permeability. In a malignant tumor microenvironment, neoangiogenesis is more active and intense, and myriads of tiny leaky vessels develop, resulting in increased blood flow and permeability [24]. Thus, the Ktrans values of malignant tumors are higher than those of benign tumors. In this study, with a fairly high temporal resolution of 5.6 s, the 90th percentile of Ktrans had the highest AUC, which was also statistically higher than the 10th percentile, probably because voxels with higher Ktrans values represent the most aggressive regions of tumors with abundant blood perfusion. This is consistent with the results of a similar Ktrans histogram analysis from another study [12]. Of the Tofts model parameters, Ktrans and the extravascular extracellular space (EES) fractional volume (Ve) are directly related to fundamental physiology and require access to the absolute values of tracer concentration, whereas Kep is the ratio of Ktrans to Ve (Kep = Ktrans/Ve), which can be derived from data regarding the shape of the tracer concentration vs time. Ktrans is more widely studied than Kep for differentiation of lesions, as the clinical value of Kep is discrepant between studies [5, 7, 25, 26]. Considering the above mentioned reasons, we chose to use Ktrans to conduct this study.

The ADC histogram analysis showed that voxels with lower ADC values had better diagnostic value than those with higher values, which is consistent with the results reported by Suo et al. [20] that the minimal ADC value had the highest AUC. This trend may be reflected by the histological heterogeneity of tumors; for instance, IDC usually consists of a variety of components such as cancer nests, stroma, intratumoral fibrosis or necrosis and intraductal components, and thus presents radiological heterogeneity on ADC maps. The 10th percentile of the ADC histogram may indicate the more invasive part of malignant breast tumors. The ADC values of breast lesions in this study were relatively lower than those reported in other study that used RS-DWI [19]. One possible reason is that we used a whole lesion VOI, which involved components such as stroma and intratumoral fibrosis that have higher ADC values than tumor nests. Another issue is that the ADC map was linearly resliced according to the Ktrans map to generate the pixelwise Ktrans-ADC map, which might have resulted in a slight decrease in ADC values.

Multiparametric imaging analysis has raised a lot of interests in recent years. In early pilot studies, the ADC was taken as a supplementary parameter to the DCE imaging to obtain a more definitive diagnosis [17, 27, 28]. However, in these studies, data were not quantified, and diagnostic accuracy was still restricted by the experience of radiologists. To develop quantitative and objective methods for interpreting data, several statistical models, such as the Local Hyperplane-based relief feature weighting scheme [29], the linear discriminant analysis [30] and the step-wise multivariate logistic regression model, [31] were used to incorporate DCE and DWI information.

Histograms depict the distribution of datasets; kurtosis measures how extreme observations are, and skewness measures the asymmetry of distribution. From our results, the kurtosis of the 1D and 2D histograms was higher (although the kurtosis of the ADC histogram revealed no significant differences) for benign lesions than for malignant lesions. This is probably because cells are more uniform and regularly aligned with balanced blood flow in benign lesions, where most of the voxels tend to crowd together, and form a sharper peak. Positive skewness is called right-skewed, and indicates that the tail on the right side is longer than that on the left side, and the mass of the distribution is concentrated on the left, making the histogram curve lean to the left. In our results, the ADC skewness of malignant tumors was positive, and that of benign lesions was negative. Thus, malignant tumor histogram curves lean to the left while benign lesion histogram curves lean to the right. This is because most of the voxels in malignant tumors have low ADC values and lie to the left side of the histogram, while voxels in benign lesion tend to lie to the right side. Similar results were also obtained in another ADC histogram analysis study [20]. If the skewness of two parameters has the same sign, the parameter with the higher absolute value is more skewed. As the results revealed, the Ktrans skewness of benign lesions was higher than that of malignant tumors, probably because the Ktrans values of benign lesions were lower than those of malignant lesions, and most voxels lie to the left, making the curve lean more to the left. Such differences might not be statistically significant in 1D histograms, as the kurtosis of ADC and the skewness of Ktrans in this study and other studies [12, 20, 32], but these differences are amplified in the 2D histogram.

This study had several limitations. First, there was misregistration between ADC map and DCE images, which could have influenced VOIs, especially for small lesions and non-mass lesions. Another limitation is that DWI was performed approximately 17 min after the administration of contrast media. Although a prior study has shown that there are no significant effects on ADC values after the administration of a gadolinium-based contrast agent [33], it may be preferable to acquire the DWI sequence before contrast injection to avoid any confounding effects. Finally, the sample size of benign lesions was relatively small. Thus, this method needs to be tested on a larger scale.

## Conclusions

In summary, the combination of Ktrans and ADC performed better than single parameters alone using histogram analysis for differentiating breast lesions. The 2D kurtosis and 2D skewness are both candidates to help characterize breast lesions.

## Abbreviations

MRI: magnetic resonance imaging
CAIPIRINHA: controlled aliasing in parallel imaging results in higher acceleration
TWIST: time-resolved angiography with stochastic trajectories
VIBE: volumetric interpolated breath-hold examination
DCE: dynamic contrast-enhanced
RS-DWI: readout-segmented diffusion-weighted imaging
VOI: volume of interest
ADC: apparent diffusion coefficient
Ktrans: volume transfer constant
AUC: area under the curve
BI-RADS: Breast Imaging Reporting and Data System
ROC: receiver operating characteristic
IDC: invasive ductal carcinomas
DCIS: ductal carcinomas in situ
ILC: invasive lobular carcinoma
SPC: solid papillary carcinoma
MEC: myoepithelial carcinoma
IP: intraductal papillomas
